# Capuchin and rhesus monkeys but not humans show cognitive flexibility in an optional-switch task

**DOI:** 10.1038/s41598-019-49658-0

**Published:** 2019-09-13

**Authors:** Julia Watzek, Sarah M. Pope, Sarah F. Brosnan

**Affiliations:** 1 Department of Psychology, Language Research Center, Georgia State University, Atlanta, GA USA; 2 Neuroscience Institute, Georgia State University, Atlanta, GA USA; 3 Department of Psychology, University of Texas at Austin, Austin, TX USA; 4 Department of Philosophy, Center for Behavioral Neuroscience, Georgia State University, Atlanta, GA USA

**Keywords:** Evolution, Decision, Problem solving, Psychology

## Abstract

Learned rules help us accurately solve many problems, but by blindly following a strategy, we sometimes fail to find more efficient alternatives. Previous research found that humans are more susceptible to this “cognitive set” bias than other primates in a nonverbal computer task. We modified the task to test one hypothesis for this difference, that working memory influences the advantage of taking a shortcut. During training, 60 humans, 7 rhesus macaques, and 22 capuchin monkeys learned to select three icons in sequence. They then completed 96 baseline trials, in which only this learned rule could be used, and 96 probe trials, in which they could also immediately select the final icon. Rhesus and capuchin monkeys took this shortcut significantly more often than humans. Humans used the shortcut more in this new, easier task than in previous work, but started using it significantly later than the monkeys. Some participants of each species also used an intermediate strategy; they began the learned rule but switched to the shortcut after selecting the first item in the sequence. We suggest that these species differences arise from differences in rule encoding and in the relative efficiency of exploiting a familiar strategy versus exploring alternatives.

## Introduction

As humans, we live in complex environments and inevitably have to rely on imperfect information when we make decisions. Searching for information takes time, cognitive resources, and can result in errors while we figure out which strategies work and which do not. But once discovered, learned rules can save us that effort while helping us solve many everyday problems, especially if the environment is predictable^1,2^. However, humans and animals live in environments that change over time and space, and different problem-solving strategies will be more adaptive in some environments than in others. When the situation changes, rules of thumb can fail and either become less efficient than other solution strategies or become altogether ineffective^3^. In some cases, it can be beneficial to use learned rules even when they are suboptimal because constraints in our cognitive system can increase the cost and decrease the benefit of using alternative strategies^4^. In this study, we consider the role of one such constraint, working memory availability, on our ability to recognize when familiar solutions may no longer be efficient and to adopt novel strategies that are more beneficial.

Currently, our understanding of such cognitive flexibility is severely limited because experimental studies overwhelmingly use forced-switch measures such as discrimination-reversal^5,6^, card-sorting^7,8^, or cued-switch tasks^9–11^ that *require* participants to switch between solution strategies. However, in real-life scenarios, we typically need to select among multiple solution strategies to make good decisions. Cognitive flexibility in these contexts is far more complex than only switching when necessary or prompted because multiple ‘correct’ solutions may still differ in efficiency or relative benefit (e.g., time spent, reward value, risk of predation). Optional-switch paradigms incorporate this complexity. For instance, Luchins^12^ presented participants with a maze task, in which they solved several mazes by using a circuitous zig-zag path but were then presented with mazes that could be solved either by the familiar (but lengthy) zig-zag path or via a shortcut. The majority of participants persisted in using the familiar path, despite its relative inefficiency. This susceptibility of humans to cognitive set, defined as the propensity for a learned approach to block the use of a better alternative, has been found across a variety of task designs^12–22^.

Cognitive inflexibility can be induced by even a single and implausible verbal suggestion (e.g., a person claiming to “use magic” can prevent people from seeing through an otherwise obvious trick)^23^ or by being instructed to use an unrelated rule for a single trial in a separate part of the experiment^24^. While familiarity with a rule due to such previous exposure can hinder exploration of alternatives, familiarity can also result from expert knowledge. Indeed, experts are not immune to cognitive set. For example, in a clever study using chess configurations, the availability of a well-known familiar solution prevented expert players from finding the more optimal strategy and lowered their problem-solving performance to that of players three standard deviations lower in skill level^15^ (an enormous decrease). This can affect important decisions we encounter in real life. Experts may make mistakes because they rely on well-learned procedures in seemingly familiar situations (in which it does typically result in good outcomes) even when others may be more adequate. For example, this could contribute to doctors unwittingly misdiagnosing uncommon diseases that present with common symptoms. In order for us to make good decisions, it is important to identify the conditions in which we fail to seek or adopt new strategies.

Why we show this inability to look for better solutions once we have found an adequate one remains somewhat elusive, but cross-cultural evidence suggests that cognitive flexibility varies both within and between human populations^25–28^. Cultural influences may change how people conceptualize the problem and therefore how flexibly they approach it^25^. However, cultures differ on many dimensions that may also affect cognitive flexibility, e.g., language, the degree of formal education, and the complexity and predictability of people’s social and physical environment. For example, schooling in Western cultures may encourage blind repetition and reinforce the idea that a single correct solution exists for a given problem^29^.

One way to isolate the underlying mechanisms that lead to the cognitive set bias is to test the extent to which it is present in other species that lack human language and culture. Doing so helps us understand whether the processes behind the bias are unique to humans or shared with other animals. This, in turn, gives us insight into why it may have evolved and potentially highlights different ways to solve the same problem. To compare susceptibility to cognitive set in humans and other primates, Pope and colleagues^30^ created the LS-DS task, a nonverbal optional-switch task that uses a three-step sequence as the learned strategy (LS). In this study, two out of four squares briefly lit up on a computer screen, and participants learned to copy this computerized demonstration to select the same two squares in sequence. If done correctly, a blue triangle was revealed and could be selected to obtain a reward. Thus, the learned strategy consisted of Square 1 → Square 2 → Triangle. After participants became proficient in this strategy, they were given trials in which either the learned strategy or a more efficient, direct strategy (DS, or shortcut) could be used. In these probe trials, the triangle appeared at the same time as the demonstration. Participants could either use the learned strategy by attending to the demonstration and by subsequently performing the three-step sequence or they could use the direct strategy by immediately selecting the triangle. Thus, use of the alternative strategy in the LS-DS task is optional, but it is more efficient in that it saves time and is less prone to error.

Baboons (*Papio papio*) used the direct strategy immediately and in 99% of trials, yet only 7.5% of humans used the shortcut in more than 5% of trials^30^. In other words, the humans, but not the baboons, were impaired by cognitive set on the LS-DS task. Chimpanzees (*Pan troglodytes*) also regularly evoked the shortcut, but they also used a partial shortcut by selecting the first square, but skipping the second square, and instead selecting the triangle (the switch strategy, SS)^31^. One interpretation of these findings is that differences in initial rule-encoding or sequential processing impact primates’ response styles. Specifically, humans’ ability to conceptualize the problem verbally in just a handful of trials may result in more firmly encoded rules, making them less likely to use the alternative, direct strategy. Baboons and chimpanzees, on the other hand, required tens of thousands of trials to pass training, suggesting that the rule was difficult for them to learn and may have been less well encoded even after training. A weakly encoded learned strategy, in turn, may have been easier to replace with the shortcut.

Here we explore an alternative explanation. We suggest that differences in working memory capacity might influence strategy use on the LS-DS task. Specifically, limited working memory capacity or increased load might promote the use of the shortcut, which requires no working memory, over the use of the learned strategy, which requires participants to maintain the order and locations of Square 1 and Square 2 online. What we know of these species provides support for this; while baboons exhibit aspects of serial recall that resemble that of humans, in a direct comparison they achieved a lower overall capacity for the number of items remembered^32^. Indeed, memory in other primates appears to be similarly limited compared to humans^33–36^ (but see ref.^37^).

Further support comes from studies of humans with differing working memory capacities. When solving math problems, people with higher working memory tended to stick to a complicated learned rule, whereas people with lower working memory availability were better able to adopt a simple alternative^38^. One possibility is that the complicated learned rule imposed a greater cognitive strain on participants with lower working memory, and thereby promoted use of a simpler alternative. Conversely, participants with higher working memory were presumably less constrained by the complexity of the learned rule and would not benefit as greatly from switching to the simple strategy. Indeed, when placed under stress, the higher working memory participants were just as likely to use the simple strategy as the lower working memory participants. Thus, use of the simple alternative increased with the relative benefit of using it.

Interestingly, work with other species indicates that the relationship between working memory and flexibility may not be so straightforward. In another recent comparative study of cognitive flexibility, pigeons persevered whereas both children and adults chose more flexibly^39^. The authors suggest that the pigeons’ limited working memory may have hindered them from switching effectively in their forced-switch paradigm. Here we explore whether the reverse may be true in an optional-switch task, that is, whether limited working memory might encourage rather than hinder flexible strategy use when more than one solution strategy is available, especially if one is less demanding than another.

In the current study, we aimed to assess the impact of working memory on shortcut use in the nonverbal LS-DS task. We modified the task so that the learned strategy no longer required participants to attend to a demonstration nor remember the positions of Square 1 and Square 2. Participants still had to select the squares in sequence, but instead of appearing and disappearing again in quick succession, both squares were present from trial start and remained there until participants selected them. Thus, participants no longer needed to track the squares’ locations and hold them in working memory to succeed. We predicted that reducing the working memory requirements in the LS-DS task would suppress shortcut use because the learned strategy would be similarly cognitively demanding as the shortcut, albeit still less efficient.

Further, we wanted to test whether the apparent advantage of non-human primates over humans in this task extended to New World monkeys and another Old World monkey species. The ability to flexibly respond to changing environmental conditions is crucial to survival and may relate to factors such as phylogeny, feeding ecology, or social structure^40^. Here, we measured capuchin monkeys’ (*Cebus [Sapajus] apella*), rhesus macaques’ (*Macaca mulatta*), and human adults’ performance on this modified, easier LS-DS task (the EZ LS-DS) to gain an understanding of how species differences in working memory availability interact with shortcut use within the primate lineage. Features of the monkeys’ ecology (such as their reliance on ephemeral fruit sources^41^ or extractive foraging using tools^42^) suggest that they are well adapted to fluctuating environments. Further, while both capuchins and macaques can successfully change their behaviour in some contexts (e.g., depending on the presence of others or the utility of tools^43–46^), rhesus, but not capuchins, showed flexible information-seeking behaviour in a previous task^47^. Thus, both similarities and differences in this optional-switch task would inform our understanding of the phylogenetic and ecological distribution of cognitive flexibility. We hypothesized that the two monkey species would behave more like humans and increasingly use the learned strategy (rather than the shortcut) when working memory requirements are alleviated.

## Methods

### Participants

We recruited 60 undergraduates from Georgia State University’s SONA participant pool (50 female, 10 male; age *M* ± *SD* = 19.8 ± 3.9 years, range: 19–39 years; 15.0% Hispanic; 51.7% Black/African American, 20.0% Asian, 18.3% Caucasian/White, 5.0% more than one race, 5.0% declined to answer). As is standard for this participant pool, students received course credit for their participation in the study no matter how they performed or how long they participated. We also tested 22 capuchin monkeys (16 female, 6 male, age: *M* ± *SD* = 16.09 ± 8.13, range: 6 to ca. 42 years) and 7 rhesus macaques (all male, age: *M* ± *SD* = 21.00 ± 6.95, range: 15–35 years) at the Language Research Center at Georgia State University.

Capuchin monkeys were socially housed in mixed-sex groups in indoor/outdoor enclosures with a variety of climbing structures, visual barriers, and regularly provided enrichment devices (e.g., foraging boards and puzzle boxes). Capuchins had been trained to separate voluntarily into attached testing boxes for cognitive and behavioural studies. They were never required to come into the test boxes for testing, and they could choose not to participate at any time. Rhesus monkeys were individually housed with continuous auditory and visual access to other monkeys and, when possible, regular social periods with compatible partners. Their enclosures doubled as testing boxes, but they too could choose not to participate at any time. Participants had *ad libitum* access to water, including during testing, and are never food deprived (except for medical reasons unrelated to research studies). All testing food was given in addition to their daily diet of vegetables, fruit, and primate chow.

This study was purely behavioural, non-invasive, and was carried out in accordance with all applicable international, national, and institutional ethical guidelines and legal requirements. All procedures were approved by the Georgia State University Institutional Review Board (IRB H18085) and the Institutional Animal Care and Use Committee (IACUC capuchins: A16031, rhesus: A16030). Georgia State University is fully accredited by the Association for Assessment and Accreditation of Laboratory Animal Care (AAALAC).

### Procedure

Monkeys were tested in individual test boxes using a computer testing system in which they made their choices by moving the onscreen cursor with a joystick (described in detail in ref.^48^). Monkeys could complete as many trials as they wanted during their test sessions and were tested repeatedly on different days until they completed testing.

Humans gave informed consent and were tested individually in a computer laboratory. They received minimal instructions, in which the experimenter demonstrated the correct and incorrect feedback screens to them and stated that they would move the cursor by using the arrow keys on a standard keyboard and should try to work as fast and accurately as possible. Humans were tested in a single session until they completed testing, but no longer than 60 minutes.

### The EZ LS-DS task

We tested participants on a simplified version of the LS-DS task^25,30,31^ designed to be less reliant on working memory. Participants first completed three training phases to learn the rule before moving on to testing.

Participants made their selections by moving the cursor into contact with the stimuli. The cursor was returned to the centre of the screen after each selection. All trials began with a start screen. After selecting the start box, the response screen was presented. Following correct responses, participants received positive auditory feedback (*whoop*), and monkeys additionally received a banana-flavoured food pellet. Following incorrect responses, participants received negative auditory and visual feedback (*buzz* and a green screen) and a two-second timeout. All trials were followed by an inter-trial interval of one second (in addition to the timeout, if applicable).

#### Training phase

During training, monkeys completed blocks of 24 trials, and humans completed blocks of 8 trials. For each trial, we randomized the locations of the stimuli, with the constraint that each spatial combination occurred an equal number of times within a trial block. Participants automatically advanced to the next phase if they reached at least 80% accuracy in two separate trial blocks.

In Training 1, participants saw a striped square (Square 1) and a dotted square (Square 2) and were rewarded for first selecting Square 1 and then Square 2. In Training 2, we increased the number of response options so that two blank squares were present in addition to Squares 1 and 2. In Training 3, upon selection of Squares 1 and 2, a blue triangle appeared, replacing one of the two blank squares. Participants were rewarded when they selected the triangle (Square 1 → Square 2 → Triangle, i.e., the learned strategy).

Four humans failed to pass Training 1 during their 60-minute testing session. One male capuchin monkey failed to pass Training 1, and one female capuchin stopped participating in the experiment during Training 1. Additionally, one female capuchin initially stopped participating after passing Training 2. We reset her program to require her to reach criterion in a third Training 2 trial block, after which she went on to finish the experiment.

Capuchins completed Training after *Mdn* = 2316 trials, *IQR*: 1338–4716, range: 576–7368 (*Mdn* Training 1: 1488, Training 2: 612, Training 3: 252). Rhesus macaques completed Training after *Mdn* = 1008 trials, *IQR*: 732–1932, range: 288–4320 (*Mdn* Training 1: 696, Training 2: 192, Training 3: 72). Humans completed Training after *Mdn* = 56 trials, *IQR*: 56–64, range: 48–208 (*Mdn* Training 1: 24, Training 2: 16, Training 3: 16).

#### Test phase

Participants who passed training completed 4 blocks of 48 test trials. Each block consisted of 24 BASE trials and 24 PROBE trials in random order, resulting in a total of 96 BASE trials and 96 PROBE trials. For each trial, we randomized the locations of the stimuli, with the constraint that each spatial combination occurred an equal number of times per block.

**Figure 1:**
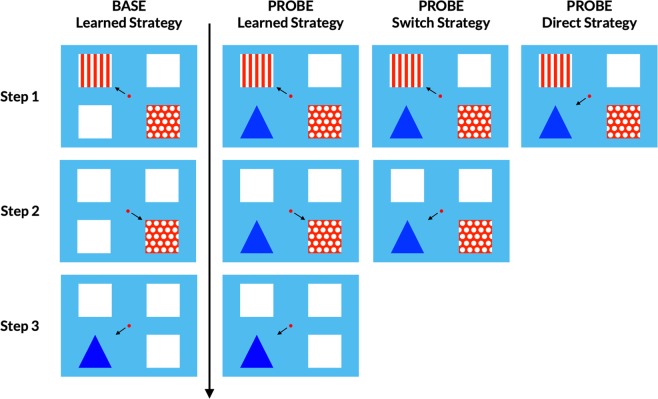
Schematic trial progression. Three different strategies constitute a correct response in BASE and PROBE trials. Arrows indicate the icon to be selected.

BASE trials were identical to Training 3 (Fig. 1), except that the triangle was “hidden” behind one of the blank squares from the beginning. That is, selecting that blank square was recorded as a correct response to establish a baseline measure of accidental shortcut use. In PROBE trials (Fig. 1), the triangle was visible from the beginning and could be selected directly for a more immediate reward (the direct strategy). Alternatively, participants could continue to use the learned strategy, Square 1 → Square 2 → Triangle, to produce a reward. Participants could also switch to the shortcut midway through the LS sequence (the switch strategy, Square 1 → Triangle).

### Data analysis

To calculate a “true” measure of DS use, we subtracted the number of BASE trials in which participants used the direct strategy (a measure of accidental DS use) from the number of PROBE trials in which they used the direct strategy. Such accidental DS use occurred on average in only 1.8% of trials. Following previous studies^25,30^, we first classified participants as DSers if they used the direct strategy in more than 5% of trials (after correction) and used a chi-square test to assess whether species differed in the number of DSers.

We used logistic mixed-effects models with a binomial error structure to further analyse shortcut use and two measures of accuracy (binomial variables). We included participant identity as a random effect in all models to account for different baseline rates of the dependent variables. We used the *lme4* package^49^ in R 3.5.1^50^ to fit the models, likelihood ratio tests using single-term deletions to assess the test predictors’ importance, and the *emmeans* package^51^ to compute pairwise contrasts with the Tukey correction for multiple comparisons.

To determine whether species differed in how consistently they used the shortcut over time, we analysed the proportion of trials in which the direct strategy was used. As fixed effects, we included species, test block, and their interaction. We further included training duration (the number of trials required to pass criterion) as a covariate to assess whether difficulties with acquiring the learned strategy affected shortcut use.

To determine whether species differed in their performance, we analysed the proportion of trials that were overall correct (i.e., trials in which participants ultimately selected the triangle and were rewarded, regardless of strategy). As fixed effects, we included species, trial type (BASE vs. PROBE), and their interaction.

To determine whether species differed in whether they incurred switch costs, we analysed any deficits in accuracy related to switching between the learned strategy and the direct strategy. We compared BASE trial accuracy following a BASE trial in which the learned strategy was used (BASE LS) to BASE trial accuracy following a PROBE trial in which the direct strategy was used (PROBE DS). As fixed effects, we included species, trial type of the preceding trial, and their interaction. We did not compare PROBE trial accuracy when sticking with the direct strategy (from PROBE DS) or switching to the direct strategy (from BASE LS) because using the shortcut always resulted in 100% accuracy, regardless of the trial type and strategy in the previous trial.

## Results

### Strategy use

**Figure 2:**
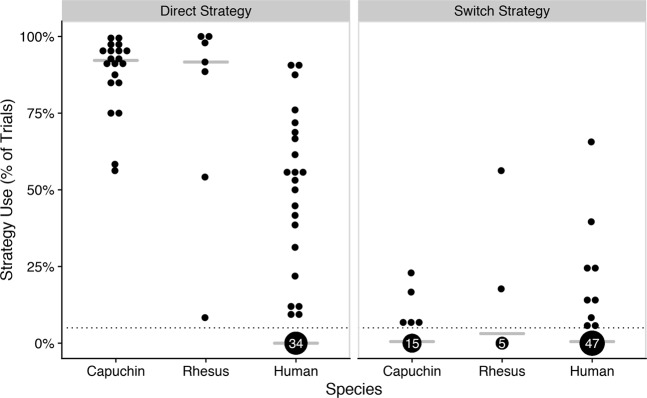
Strategy use. Percentage of PROBE trials in which the direct strategy (left) and switch strategy (right) were used by species (corrected for accidental shortcut use in BASE trials). Dotted line represents 5%. Above 5%, each point represents one participant; below 5%, point size and labels represent the number of participants. Each participant is shown twice, once in each panel. Crossbars represent medians.

All 20 capuchin monkeys and all 7 rhesus macaques used the direct strategy in more than 5% of trials, whereas a significantly smaller proportion (39%) of humans did so (Fig. 2, left), χ^2^(2) = 27.77, *p* < 0.001. In fact, 70% (*n* = 14/20) of capuchins and 71% (*n* = 5/7) of rhesus macaques used the direct strategy the very first time it was available, and 20% (*n* = 4/20) of capuchins and 29% (n = 2/7) rhesus monkeys used it every single time it was available. In stark contrast, only one human (2%) used the shortcut the first time it was available, and none used it every single time. Indeed, all capuchin and rhesus monkeys had used the shortcut by PROBE trial number 8, whereas the humans that used the shortcut at all first did so by PROBE trial number *Mdn* = 43 and as late as PROBE trial number 91 (out of 96 total).

**Figure 3:**
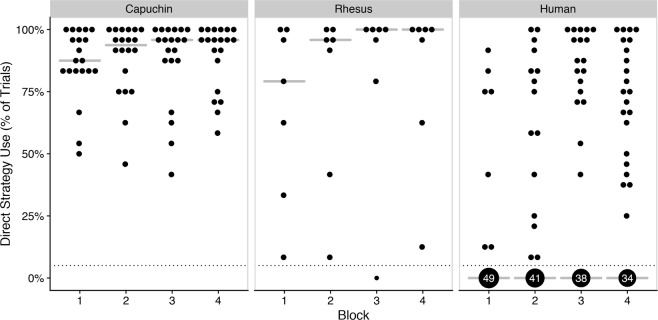
Shortcut use over time. Percentage of PROBE trials in which the direct strategy was used across the four testing blocks by species (corrected for accidental shortcut use in BASE trials). Dotted line represents 5%. Above 5%, each point represents one participant; below 5%, point size and labels represent the number of participants. Crossbars represent medians.

On a more detailed level, we found a significant species × block interaction effect on how often the shortcut was used, χ^2^(2) = 169.47, *p* < 0.001. Humans used the shortcut in significantly fewer trials than both capuchin, *Z* = −22.59, *p* < 0.001, and rhesus monkeys, *Z* = −21.26, *p* < 0.001. However, humans and rhesus monkeys (to a smaller extent) used the shortcut more over time, whereas capuchin monkeys did not further increase their already high shortcut use (Fig. 3). After the first block, only 13% (n = 7/56) of humans had used the shortcut in more than 5% of trials, which increased to 27% (n = 15/56) by end of block 2, 32% (*n* = 18/56) by end of block 3, and finally 39% (*n* = 22/56) by end of block 4.

There was no significant effect of training duration (the number of trials a participant required to reach the test phase) on shortcut use, χ^2^(1) = 0.568, *p* = 0.451.

Interestingly, some participants from each species used an intermediate strategy, in which they started with the learned strategy but then took the shortcut (Square 1 → Triangle). The species did not differ significantly in the number of participants that used this switch strategy (Fig. 2, right): 25% (*n* = 5/20) of capuchin monkeys, 29% (*n* = 2/7) of rhesus macaques, and 16% (*n* = 9/56) of humans did so in at least 5% of trials, χ^2^(2) = 1.18, *p* = 0.555. Except for two humans, all participants who used this switch strategy in at least 5% of trials also used the full shortcut (direct strategy) in at least 5% of trials.

### Accuracy

**Figure 4:**
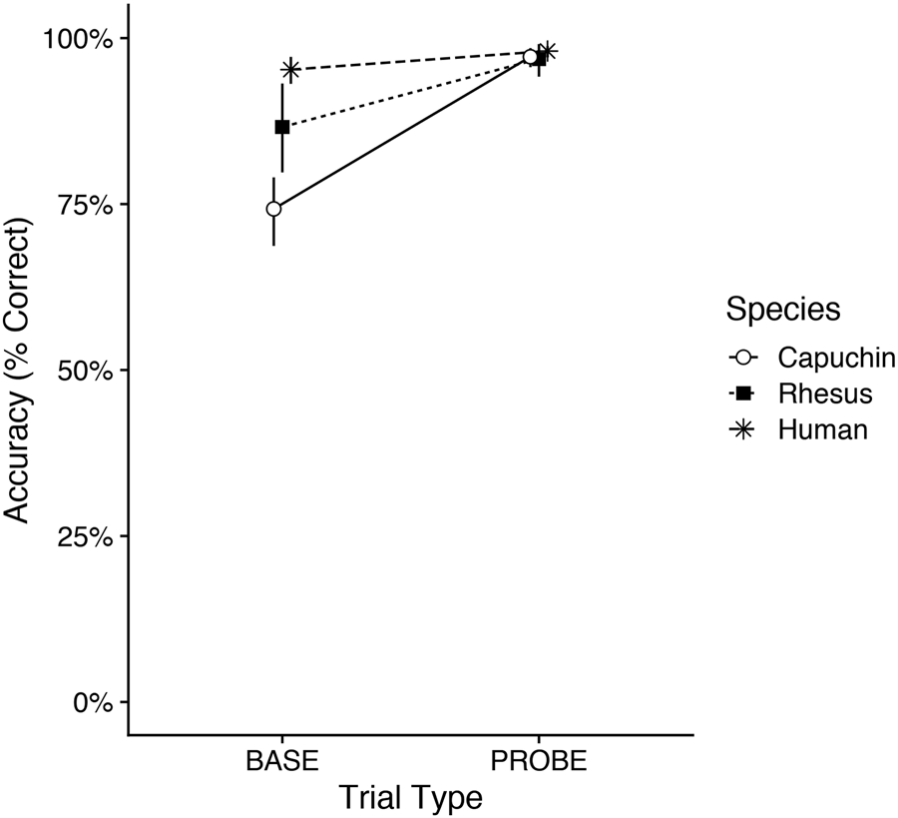
Accuracy. Mean accuracy by species and trial type. Error bars represent 95% confidence intervals.

Overall, all species performed at high levels and consistently above chance (Fig. 4; chance level for LS: ¼ × ¼ × ¼ = 2%, DS: ¼ = 25%). Humans (*M* ± *SD* = 97 ± 5% correct) typically outperformed capuchin monkeys (*M* ± *SD* = 86 ± 6% correct), and rhesus monkeys fell between (*M* ± *SD* = 92 ± 6% correct).

We found a significant species × trial type interaction effect on accuracy (Fig. 4), χ^2^(2) = 71.84, *p* < 0.001. In PROBE trials, all species performed at ceiling and did not differ in their accuracy, all *p*s > 0.05. However, all species performed worse in BASE than PROBE trials, all *p*s < 0.01. Further, capuchins did significantly worse than humans in BASE trials, *Z* = 4.96, *p* < 0.001, and rhesus monkeys fell midway between but did not differ significantly from either capuchins, *Z* = −2.30, *p* = 0.056, or humans, *Z* = 2.06, *p* = 0.099 after correcting for multiple comparisons.

Looking more closely at the learned strategy in BASE trials, we found that the capuchin and rhesus monkeys’ lower BASE trial performance was mainly driven by mistakes in selecting the first square in the sequence and, for the capuchins, also to some extent in selecting the second square (see Supplementary Information).

### Switch costs

**Figure 5:**
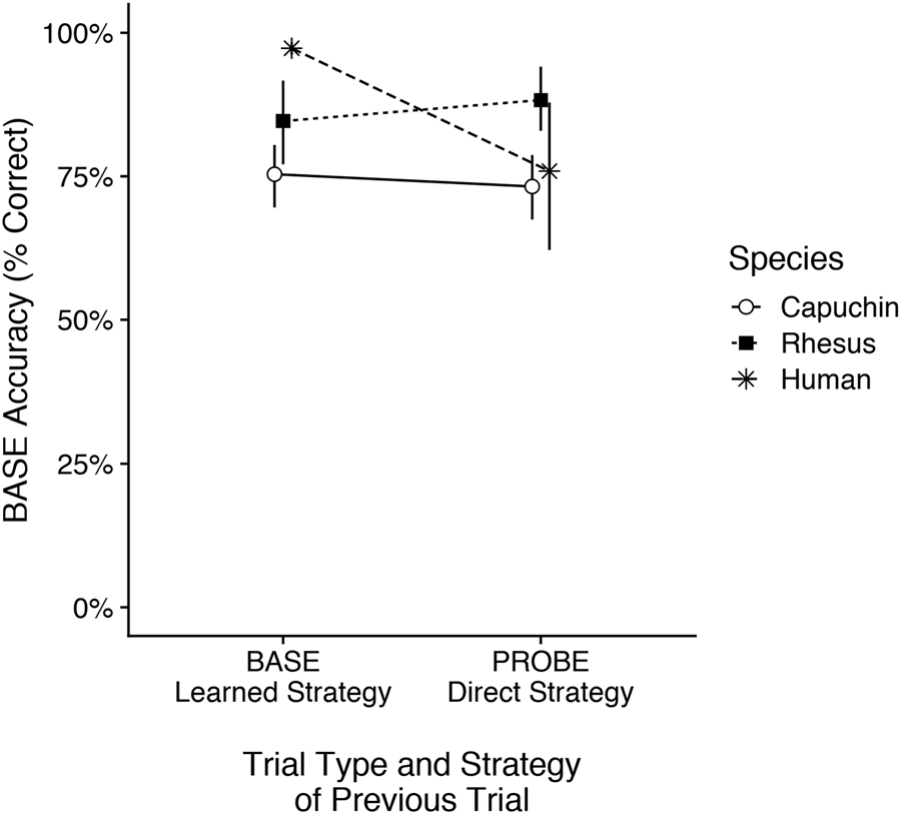
Switch costs in accuracy. Mean accuracy in BASE trials by species and trial sequence. Error bars represent 95% confidence intervals.

Our switch cost analysis revealed an interaction between species and previous trial type on BASE trial accuracy (Fig. 5), χ^2^(2) = 57.82, *p* < 0.001. Humans (*Z* = 4.11, *p* < 0.001), but neither capuchin (*Z* = 0.79, *p* = 0.430) nor rhesus monkeys (*Z* = −0.90, *p* = 0.367) performed significantly worse in BASE trials when they switched from a PROBE trial (having just used the direct strategy) than when they had just used the learned strategy in another BASE trial. As expected, all species completed PROBE trials using the shortcut faster than they completed BASE trials using the learned strategy. However, neither humans nor the two monkey species showed differences in response times when switching trial types (see Supplementary Information).

## Discussion

In this study, we assessed the ability of three primate species to break a cognitive set bias in order to use a short cut. We found that capuchin and rhesus monkeys successfully used the shortcut at high rates, soon after it first became available. In doing so, they join the ranks of baboons and chimpanzees in outperforming humans, who tend to stick with the less efficient but familiar learned strategy (i.e., they show a cognitive set bias). Furthermore, using the shortcut was indeed more beneficial, as it both increased accuracy and decreased response times in all species.

We had two rationales for this study. First, we tested the extent to which the previously reported advantage of non-human primates over humans on the LS-DS task extended to New World monkeys and another species of Old World monkey. Second, we tested the hypothesis that lower working memory requirements would increase use of the learned strategy. For this study, we used the modified EZ LS-DS task, in which the Square 1 and Square 2 stimuli were presented at the same time and remained on the screen throughout the trial, rather than appearing briefly and then vanishing, as in the original LS-DS task. Therefore, participants did not need to track stimuli locations nor hold them in working memory in order to succeed. In support of this point, capuchins and rhesus in this study required only a fraction of the training trials that the baboons and chimpanzees needed in the original LS-DS task to learn the rule. However, decreasing the working memory load required by the learned strategy did not make the monkeys more likely to use it when the shortcut was also available.

Although our monkeys did not use the full learned strategy in PROBE trials, we found that 25–30% of monkeys used the switch strategy, in which they began with the learned strategy by selecting the first square, but then took the shortcut (instead of continuing the sequence by selecting the second square). Use of this intermediate strategy suggests a shift toward more habitual rule use, perhaps enabled by the decreased working memory load. Baboons in the original LS-DS very rarely used this intermediate strategy, whereas chimpanzees did so frequently^31^. Indeed, the reported working memory capacity in baboons^32^ has been lower than that reported for chimpanzees^37^ (but see ref.^52^), though a direct comparison is lacking. We hypothesize that both species would increasingly use the switch or learned strategy when working memory requirements are alleviated (e.g., when testing them on the EZ LS-DS). However, higher working memory availability alone cannot explain the rigid inflexibility that humans show in this optional-switch task.

Another possibility is that differences in primates’ initial rule encoding affect their susceptibility to cognitive set. Although the rule in our task was much easier to learn for the monkeys than it was in the original task, they still required substantially more training than the humans, who typically picked up the rule in just a few trials. Humans’ ability to encode the rule verbally may help them learn and use the strategy much more quickly than other primates can. However, such verbally encoded rules may be more firmly rooted and therefore less likely to be replaced by alternative strategies. Further, it is thought that more cognitive effort is required to switch to and from firmly encoded rules^11^. In line with this interpretation, we found that humans, but neither of the two monkey species, exhibited switch costs in this study. They made more mistakes when using the learned strategy after just having used the shortcut.

Capuchin and rhesus monkeys, on the other hand, needed more training to meet criterion for the learned strategy and made more mistakes when using it in BASE trials. In other words, the rule was not as easy to learn or use for the monkeys, suggesting that their initial rule encoding may have been weaker. This may have allowed them to adopt the more efficient alternative strategy more readily. Indeed, 70% of the monkeys (but only a single human) used the shortcut on the very first trial it became available. In this study, we had set the criterion for training at 80% accuracy in two trial blocks. It is possible that extended training with the rule would lead to more habitual rule use and a decreased ability to break cognitive set (as it does in humans^18^). Future studies should investigate this possibility, although we note that we found no effect of training duration (number of trials until criterion was reached) on shortcut use.

Interestingly, humans started using the shortcut at higher rates than in previous studies, but only as testing progressed. To our knowledge, this is the first study to demonstrate this effect. This result is incompatible with the idea that greater working memory availability makes the shortcut less beneficial because 1) humans used it more rather than less in the EZ LS-DS and 2) working memory requirements did not change over time. Instead, we suggest that this result highlights that cognitive flexibility is a balancing act between exploitation and exploration. On the one hand, if solution strategies are so entrenched that new information is ignored, they can lead us to make inefficient decisions and miss opportunities. On the other hand, if strategies are too susceptible to new input and easily replaced, we may get distracted by irrelevant or maladaptive information.

Our results therefore fit nicely into the variability-stability-flexibility pattern of cognitive flexibility^53^. According to this framework, initial strategy selection follows a variable pattern as a result of trial-and-error learning (e.g., the training phase in the present study), but is then replaced by a stable response strategy (e.g., the learned strategy was acquired and is being used consistently). Finally, people may enter a flexible state in which they can seek and adopt alternative strategies that better meet current demands. Thus far, the framework has focused on developmental trajectories of cognitive flexibility. For example, Gopnik and colleagues^54^ found that younger children outperformed adolescents and adults on a non-social task because they were more likely to try different strategies (variability) than older participants, who preferred a familiar solution (stability). This perhaps counterintuitive developmental result nicely parallels our cross-species findings that other primate species consistently outperform humans on the Standard and EZ LS-DS task.

Transition into the flexible state can result from better executive functioning (e.g., due to development or individual differences) or it can be induced externally (e.g., by a prompt to try something new^25^ or by a change in mindset^24^). In our study, the humans who started to use the shortcut more over time became more flexible without such external prompts, possibly due to increased exposure to and familiarity with the task. We observed this change in strategy use within the same individuals and over the course of a single test session. It is reasonable to assume that these individuals’ working memory capacities stayed essentially constant during that time, and we know that the working memory requirements of the task remained the same. This result can therefore not be attributed to differences in working memory load or individual differences in executive function in general. To our knowledge, this is one of the first studies to provide within-participant evidence for the variability-stability-flexibility pattern.

One possibility is that the relative duration of these three stages varies across development, species, or cultures. Different environments may favour either more stable or more flexible problem-solving strategies. For example, unpredictable environments and limited resource availability (e.g., due to a reliance on ephemeral fruit patches or certain foraging techniques, such as those requiring tool use) may require a willingness to seek out and try alternative strategies, i.e., increased cognitive flexibility. Future research could address the potential effect of a risky environment in several ways through cross-cultural research (e.g., populations with different foraging and farming practices), comparative research (e.g., species with different feeding or social ecologies), or even within the same population (e.g., by making the rewards for different strategies probabilistic).

Good decision-making requires that we recognize when the familiar strategies that we have been exploiting may no longer be the most efficient and when we should instead explore whether other strategies may be more beneficial. In this study, use of the shortcut was both faster and boosted the monkeys’ accuracy (compared to BASE trials, in which they could only use the learned strategy), perhaps creating an incentive to adopt the shortcut early and consistently. In contrast, humans already performed at ceiling with the learned strategy (i.e., there was less of a benefit to using the shortcut) and their accuracy dropped when they did switch strategies (i.e., there was a higher cost to using the shortcut). However, over time, our human participants did begin to explore and use the alternative strategy more. Extended use of the same learned strategy may have made the time savings of the shortcut more attractive (as reduced response times can add up, and the students in our sample were motivated to work quickly) or may encourage tendencies to explore other options in general, perhaps due to boredom.

Consider the following example. Calculating the mean of five numbers by hand is fairly simple, and you can do so many times in a row without problem. Eventually, however, this would get old, and you might look for alternatives and discover the mean function in a statistics program, which allows you to do the calculation more efficiently. On the other hand, calculating an ANOVA by hand is more difficult to learn, more effortful to do correctly, and more prone to errors. In this case, you might try a different strategy as soon as it becomes available in case it is easier. We believe this nicely illustrates the situation for the humans and monkeys in this study, respectively.

Our findings suggest that, contrary to our initial hypothesis, differences in rule encoding and in the relative costs and benefits of the available strategies better explain the observed results than differences in working memory among species. However, we believe there is room for working memory to explain some of the variability in cognitive flexibility *within* species (e.g., ref.^38^). Future comparative research should expose the same individuals to different conditions that vary in their working memory requirements. We would expect low working memory load to favour use of the learned strategy and increased load to favour shortcut use (e.g., in the LS-DS task, this could be achieved by presenting more squares and requiring longer sequences to be remembered). Another promising avenue for comparative research would be to assess optional-switch cognitive flexibility and individual differences in executive functioning, such as working memory, at the same time.

In humans, of course, executive functioning typically increases with age. However, it can be difficult to tease apart its effect on cognitive flexibility in developmental studies because other factors such as knowledge and experience with formal schooling also increase with age. In Western cultures, for example, standardized testing and formal schooling may encourage rote repetition and search for a single correct solution^29^, which could stifle flexible problem-solving from an early age. However, in different studies using the original LS-DS task with the same participant pool, half the sample continued to use the learned strategy even when told explicitly “Don’t be afraid to try something new,”^25^ and about 30% did so even after watching a video demonstrating the shortcut^55^. Thus, to some extent, Westerners might stick to the learned strategy because it is what they believe they “should” do, but that is only part of the story.

One advantage of the nonverbal LS-DS task is that it facilitates research across many different populations (e.g., different species, different developmental stages, different cultures). We encourage future research in this area to systematically explore how the degree of formal education and teaching style may affect flexible strategy use independent of individual differences in executive functioning. For example, in a sample of Zoo Atlanta visitors, children (7–10 years) were at least four times more likely to use the shortcut than adults, but still more than half of them continued to use the learned strategy^30^. Similarly, in a cross-cultural study with the seminomadic Himba of Namibia, 60–70% of participants failed to adopt the shortcut^25^. They did use it more than Western undergraduates, suggesting that humans’ susceptibility to cognitive set is not universal. However, in no human sample to date have participants used the shortcut nearly as much, as early, or as consistently as any of the non-human species.

Taken together, our results suggest that a lower working memory load may facilitate initial habitual strategy use to some extent (reflected in the monkeys’ use of the switch strategy). However, working memory availability alone does not explain humans’ initial inflexibility, nor does it explain why humans increasingly used the shortcut over time. We suggest that differences in how firmly the learned strategy may have been encoded better explains the observed inter-species variation in susceptibility to cognitive set. Further, it will be important to consider differences in the relative costs and benefits of exploiting a familiar strategy versus exploring alternative strategies, and how they may change over time or different contexts. In doing so, we can move from assessing cognitive flexibility as merely absent or present (by asking yes or no) toward establishing which conditions favour more flexible or more inflexible decision-making (by asking when and how). Ultimately, this lets us take advantage of more efficient alternatives and will help us make better decisions.

## Supplementary information

Supplementary Information

## Data Availability

The datasets generated and analysed during the current study are publicly available at the Harvard Dataverse^56^.
