# Supplementary material for: Capuchin and rhesus monkeys but not humans show cognitive flexibility in an optional-switch task
Source: Sci Rep. 2019 Sep 13;9:13195. doi: 10.1038/s41598-019-49658-0 (PMC6744456; doi:10.1038/s41598-019-49658-0)
Supplement: Supplementary file 1 — Supplementary Information [file 41598_2019_49658_MOESM1_ESM.pdf]

SUPPLEMENTARY INFORMATION

Capuchin and rhesus monkeys but not humans show cognitive flexibility in an optional-switch task

Julia Watzek, Sarah M. Pope, Sarah F. Brosnan

SUPPLEMENTARY ANALYSES

In addition to the main analyses, we used logistic mixed-effects models with a binomial error structure to analyse conditional accuracy (a binomial variable) and linear mixed-effects models to analyse two measures of response time (continuous variables). We included subject identity as a random effect in all models to account for different baseline rates of the dependent variables. We used the *lme4* package[1] in R 3.5.1[2] to fit the models, likelihood ratio tests using single-term deletions to assess the test predictors' importance, and the *emmeans* package[3] to compute pairwise contrasts with the Tukey correction for multiple comparisons.

We analysed the conditional accuracy in BASE trials for each step in the LS sequence, given that the previous steps had been met. Specifically, we analysed the proportion of BASE trials in which Square 1 was correctly selected (Step 1), the proportion of correct Step 1 trials in which Square 2 was then correctly selected (Step 2), and the proportion of correct Step 2 trials in which the Triangle was then correctly selected (Step 3; i.e., the full LS sequence). We included species and LS step as fixed effects.

We analysed both trial response time and response time for the first response using a similar approach as for accuracy, comparing reaction times for both BASE LS and PROBE DS trials (current trial type) following either a BASE LS or PROBE DS trial (previous trial type). As fixed effects, we included species, current and previous trial type, and their interactions. We considered response times that were not within 1.5 × inter-quartile range of the first and third quartile for each subject as outliers and excluded them for this analysis (3.4% of trials). We further excluded trials that were preceded by an incorrect trial or trials that were themselves incorrect (overall response time) or trials with an incorrect first response (first response time), because we cannot assess which strategy was used in incorrect trials.

Conditional Accuracy in BASE Trials

We found a significant species × LS step interaction (Fig. S1), $\chi^2(4) = 9.66$, $p = .046$. Specifically, the capuchin and rhesus monkeys' lower BASE trial performance was mainly driven by mistakes in selecting the first square in the sequence and, for the capuchins, also to some extent in selecting the second square. All species made more mistakes in Step 1 (selecting Square 1) than in the subsequent Step 2 (selecting Square 2 after having correctly selected

Square 1) and Step 3 (selecting the Triangle after having correctly selected Square 1 and then Square 2). These were significant differences for humans (Step 1 vs. 2: $Z = -4.40$, $p < .001$, Step 1 vs. 3: $Z = -4.50$, $p < .001$) and capuchins (Step 1 vs. 2: $Z = -4.13$, $p < .001$, Step 1 vs. 3: $Z = -4.16$, $p < .001$) and trends for rhesus monkeys (Step 1 vs. 2: $Z = -2.10$, $p = .089$, Step 1 vs. 3: $Z = -2.11$, $p = .088$). Capuchin monkeys, but not humans nor rhesus (ps > .05), also made more mistakes in Step 2 than in Step 3, $Z = -2.63$, $p = .023$. Indeed, capuchins performed worse than humans in all three steps (all ps < .01) and worse than rhesus monkeys in Step 2 ($Z = -2.64$, $p = .023$), with a similar trend in Step 3 ($Z = -2.19$, $p = .073$). Humans and rhesus monkeys did not differ significantly in any of the steps (all ps > .05).

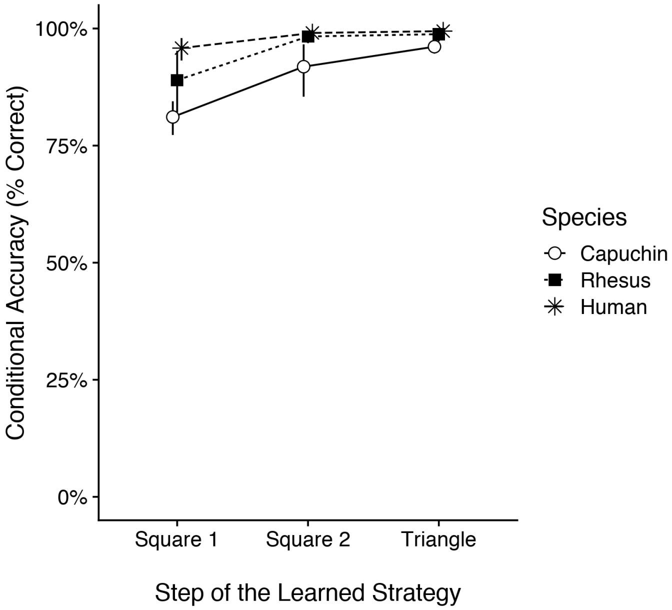

Figure S1 Conditional accuracy. Mean accuracy by species and by step of the learned strategy in BASE trials. (PROBE trials are not shown because the shortcut only involved one step.) Square 1: accuracy of selecting Square 1 on trial start, Square 2: accuracy of selecting Square 2 after having correctly selected Square 1, Triangle: accuracy of selecting the Triangle after having correctly selected Square 1 and then Square 2. Error bars represent 95% confidence intervals.

Switch Costs in Response Times

For response times when switching trial types (BASE vs. PROBE) and strategies (learned vs. direct strategy), we found a significant species × (current) trial type interaction effect (Fig. S2a), $\chi^2(2) = 61.364$, $p < .001$, but no main effect nor interactions of the previous trial's type, all ps > .05. That is, all species completed PROBE trials using the shortcut faster than they completed BASE trials using the learned strategy (all ps < .001), no matter which strategy they had used in the previous trial (i.e., BASE LS or PROBE DS). This is expected because the learned strategy requires subjects to complete more steps than the shortcut. The three species did not differ in their PROBE trial response times, all ps > .05; but in BASE trials, rhesus monkeys

responded significantly slower than both capuchins ($t(265.78) = -4.89$, $p < .001$) and humans ($t(286.36) = 4.77$, $p < .001$), who did not differ from each other ($t(265.78) = -0.90$, $p = .638$).

We used the same response time analysis for just the first response in a given trial and again found a species × trial type interaction (Fig. S2b), $\chi^2(2) = 10.15$, $p = .006$, but no main effect nor interactions of the previous trial's type, all *p*s > .05. Capuchins ($t(245.36) = -2.25$, $p = .025$) and rhesus monkeys ($t(263.27) = -3.64$, $p < .001$) but not humans ($t(985.53) = 0.22$, $p = .824$) took slightly longer to take the shortcut in PROBE trials (i.e., to select the Triangle) than to select Square 1 in BASE trials. For both trial types, rhesus monkeys were significantly slower to respond initially than both capuchins (BASE: $t(245.36) = -4.08$, $p < .001$, PROBE: $t(245.55) = -4.78$, $p < .001$) and humans (BASE: $t(263.27) = 3.45$, $p = .002$, PROBE: $t(265.68) = 4. 36$, $p < .001$), who did not differ from each other (BASE: $t(245.36) = -1.57$, $p = .259$, PROBE: $t(245.55) = -0.76$, $p = .726$).

On average, rhesus macaques responded more slowly than the other two species, both for the first choice of a trial and for the entire sequence. However, this was due to one macaque who took roughly twice as long as the other six macaques to respond.

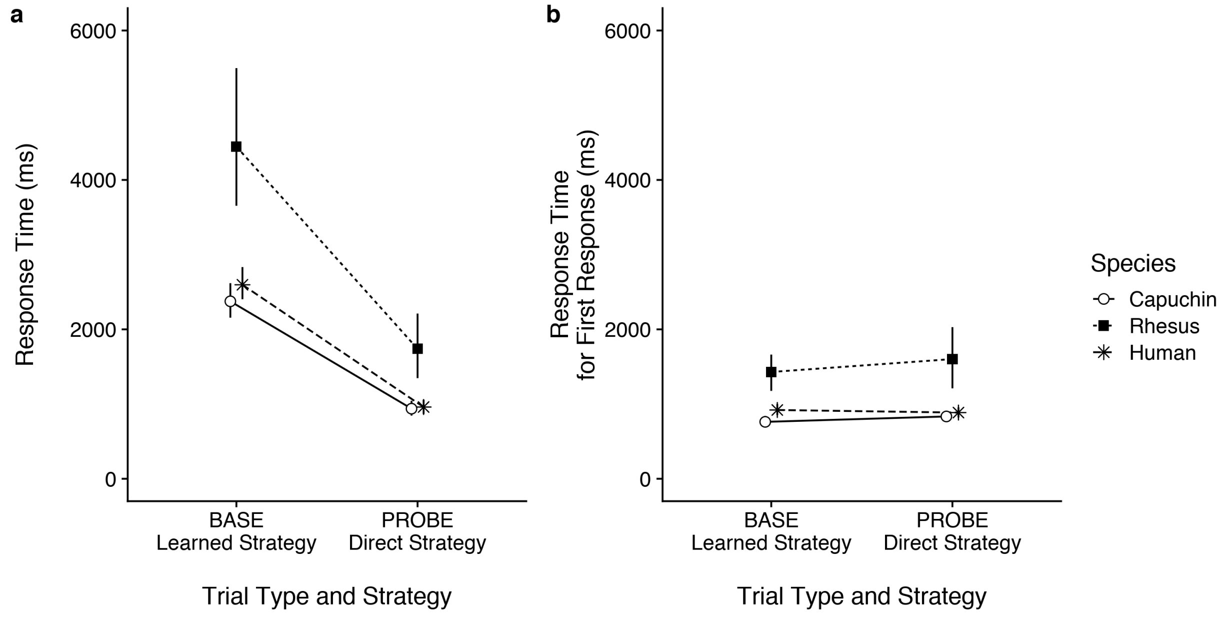

Figure S2 Switch costs in response times. (a) Mean trial response time by species and trial type. (b) Mean response time for the first response by species and trial type. Error bars represent 95% confidence intervals.

REFERENCES

1. Bates, D., Mächler, M., Bolker, B. & Walker, S. Fitting linear mixed-effects models using lme4. *J. Stat. Softw.* **67** (2015). doi:10.18637/jss.v067.i01

2. R Core Team. *R: A language and environment for statistical computing*. (Vienna, Austria: R Foundation for Statistical Computing, 2018). http://www.R-project.org/

3. Lenth, R. V. *emmeans: Estimated marginal means, aka least-squares means*. (2018). https://CRAN.R-project.org/package=emmeans
